# Bone morphogenetic proteins − 7 and − 2 in the treatment of delayed osseous union secondary to bacterial osteitis in a rat model

**DOI:** 10.1186/s12891-018-2203-7

**Published:** 2018-07-27

**Authors:** Lars Helbig, Georg W. Omlor, Adriana Ivanova, Thorsten Guehring, Robert Sonntag, J. Philippe Kretzer, Susann Minkwitz, Britt Wildemann, Gerhard Schmidmaier

**Affiliations:** 1 Clinic for Orthopedics and Trauma Surgery, Center for Orthopedics, Trauma Surgery and Spinal Cord Injury, Heidelberg University Hospital, Schlierbacher Landstrasse 200a, 69118 Heidelberg, Germany; 2 Clinic for Trauma and Orthopaedic Surgery, BG Trauma Center Ludwigshafen at Heidelberg University Hospital, Ludwig-Guttmann-Strasse 13, 67071 Ludwigshafen on the Rhine, Germany; 3 Laboratory of Biomechanics and Implant Research, Clinic for Orthopedics and Trauma Surgery, Heidelberg University Hospital, Schlierbacher Landstrasse 200a, 69118 Heidelberg, Germany; 4 Berlin-Brandenburg Center for Regenerative Therapies, Charité—Universitätsmedizin Berlin, 13353 Berlin, Germany; 5 Experimental Trauma Surgery, Universitätsklinikum Jena, 07747 Jena, Germany

**Keywords:** Animal model, Rat, Delayed osseous union, Osteitis, Biomechanical testing, Micro-CT, Bone morphogenetic protein 7, Bone morphogenetic protein 2

## Abstract

**Background:**

Bone infections due to trauma and subsequent delayed or impaired fracture healing represent a great challenge in orthopedics and trauma surgery. The prevalence of such bacterial infection-related types of delayed non-union is high in complex fractures, particularly in open fractures with additional extensive soft-tissue damage. The aim of this study was to establish a rat model of delayed osseous union secondary to bacterial osteitis and investigate the impact of rhBMP-7 and rhBMP-2 on fracture healing in the situation of an ongoing infection.

**Methods:**

After randomization to four groups 72 Sprague-Dawley rats underwent a transverse fracture of the midshaft tibia stabilized by intramedullary titanium K-wires. Three groups received an intramedullary inoculation with *Staphylococcus aureus* (10^3^ colony-forming units) before stabilization and the group without bacteria inoculation served as healing control. After 5 weeks, a second surgery was performed with irrigation of the medullary canal and local rhBMP-7 and rhBMP-2 treatment whereas control group and infected control group received sterile saline. After further 5 weeks rats were sacrificed and underwent biomechanical testing to assess the mechanical stability of the fractured bone. Additional micro-CT analysis, histological, and histomorphometric analysis were done to evaluate bone consolidation or delayed union, respectively, and to quantify callus formation and the mineralized area of the callus.

**Results:**

Biomechanical testing showed a significantly higher fracture torque in the non-infected control group and the infected rhBMP-7- and rhBMP-2 group compared with the infected control group (*p* < 0.001). RhBMP-7 and rhBMP-2 groups did not show statistically significant differences (*p* = 0.57). Histological findings supported improved bone-healing after rhBMP treatment but quantitative micro-CT and histomorphometric results still showed significantly more hypertrophic callus tissue in all three infected groups compared to the non-infected group. Results from a semiquantitative bone-healing-score revealed best bone-healing in the non-infected control group. The expected chronic infection was confirmed in all infected groups.

**Conclusions:**

In delayed bone healing secondary to infection rhBMP treatment promotes bone healing with no significant differences in the healing efficacy of rhBMP-2 and rhBMP-7 being noted. Further new therapeutic bone substitutes should be analyzed with the present rat model for delayed osseous union secondary to bacterial osteitis.

## Background

Bacterial bone infections due to trauma with subsequent delayed or impaired fracture healing are highly feared complications in orthopedics and traumatology and represent a great challenge. Open fractures in particular exhibit an incidence of osteitis as high as 55% [1]. Bacteria colonizing implants can interfere with physiological bone formation and remodeling mechanisms and lead to a higher risk of impaired fracture healing [1, 2]. In most cases, *Staphylococcus aureus* (*S. aureus*) is responsible for infection. The bacterium can infiltrate osteoblasts, builds biofilms and mutates into its small colony variants form, all leading to a high antibiotic resistance [1, 3, 4]. Therefore currently available therapeutic options must involve not only antibiotic treatment but also repeated surgical debridement, potentially exacerbating extensive bone defects [4]. Furthermore, prolonged treatment of delayed fracture healing may have a profound effect on both the medical and emotional condition of the patient as well as his financial and professional security [5].

Bone morphogenetic proteins (BMPs), already established as a useful addition to classic therapy in cases of non-union and delayed fracture union, have not been sufficiently investigated in infected fractures of the long bones [6]. Only rhBMP-2 was evaluated on surgical infections in a rabbit posterolateral lumbar fusion model showing an insignificant trend toward improved fusion rate and less mortality [7]. BMP-application is officially not indicated in the situation of an infected non-union of the long bones, because scientific data in the literature is insufficient. Currently, BMPs can only be used off-label in the situation of an infection.

BMPs are naturally responsible for the induction of osteogenic differentiation of mesenchymal stem cells and for angiogenesis and show limited expression during delayed fracture healing [8–10]. Recombinant(rh)BMP-2 and rhBMP-7 have been proven to promote fracture healing both in delayed fracture healing and non-union animal models [11–14]. RhBMP-2 has shown high potential in the treatment of open fractures in clinical trials, leading to faster union establishment, lower infection rate and lower number of surgeries needed [15–17]. Similarly, rhBMP-7 could enhance the efficacy and the treatment success of non-union in the combined therapy with autologous cancellous bone [18].

The outcome of BMP-enhanced treatment of delayed fracture healing in the situation of an ongoing low-grade bone infection has not yet been sufficiently explored.

Therefore, the goal of this study was to investigate the impact of rhBMP-7 and rhBMP-2 on fracture healing in an animal model of delayed osseous union secondary to chronic bacterial osteitis [19]. The hypothesis was that rhBMP-7 and rhBMP-2 may improve bone healing in the situation of an infection without significant differences between both factors. Primary objective was the fracture torque in biomechanical evaluations, secondary objectives were radiological and histological outcome.

## Methods

### Preparation of bacterial inoculum

Bone infection in this study was induced by the bacterium *Staphylococcus aureus subsp. aureus* Rosenbach (ATCC® 49,230™) – a strain isolated from a patient with chronic osteomyelitis and effectively used in the induction of bone infections before [20]. The needed amount of 10^3^ colony-forming units (CFU) had already been determined in a previous study [19]. Bacterial cultivation was performed according to the official product instruction, using Trypticase Soy Broth (TSB) for liquid cultures and blood agar plates for plating (Becton, Dickinson and Company, New Jersey, USA). All cultures were handled under sterile conditions and incubated under permanent oxygen supply at a temperature of 37 °C. Bacterial CFU counts on blood agar plates and spectrophotometric measurements of liquid cultures were performed to establish a calibration formula. An overnight culture of *S-. aureus* in TSB, containing 10^3^ CFU in 10 μl was used for each surgery.

### Animals and surgical procedure

All experiments were approved by the Animal Experimentation Ethics Committee of Karlsruhe (35–9185.81/G-171/11). 72 four-months old female Sprague Dawley rats (Charles River Laboratories, Germany) with an average weight of 296 g were divided into four study groups with 18 rats per group. Two of the groups were used as controls with induction of a bone infection (+*S. aureus*) or without infection (control). The animals in the two other groups were used as study groups for the treatment with rhBMP-7 (rhBMP-7 + *S. aureus*) or rhBMP-2 (rhBMP-2 + *S. aureus*).

#### Surgery preparation and follow-up

Prior to surgery, general anesthesia was performed by a weight-adapted subcutaneous injection of medetomidine (Dorbene Vet 1 mg/ml), midazolam (Dormicum 15 mg/3 ml) and fentanyl (Fentanyl – Janssen 0.05 mg/ml) after sedation with isoflurane in a sedation box. Additional preparation for surgery included shaving and disinfecting the right hind leg of the rat and covering the animal body with sterile sheets. Body temperature and anesthesia depth were tested periodically during surgery. After completion of the surgical procedure an anesthesia antidote mixture of atipamezole (Antisedan 5 mg/ml), flumazenil (Flumazenil Kabi 0.1 mg/ml) and naloxone (Naloxon Incresa 0,4 mg/ml) was injected. Clinical condition and body weight were controlled and a three-day post-operative analgesic medication with buprenorphine (Temgesic) was applied.

#### Fracture and infection model

During the first surgery all right tibiae were fractured and a bacterial infection was induced in the infection groups. A 5 mm incision through the skin and fasciae medial to the ligamentum patellae was followed by drilling a 1 mm hole through the cortical tibial bone at the proximal metaphysis to access the medullar cavity. Afterwards intramedullary reaming without irrigation was performed using a 0.8 mm k-wire up to the distal part of the medullar cavity. Tibia and fibula were then fractured using an established fracture device [12, 19]. The right hind leg was placed on a metal plate and a weight of 600 g was fixated 15 cm above the leg with a removable pin. Removal of the pin resulted in a sudden free fall of the weight and a consequent transverse fracture of tibia and fibula (AO 42-A3) with a momentum of 1.03 kg m/s. The bacterial inoculum of 10^3^ CFU / 10 μl *S. aureus* was injected with a microliter syringe into the medullar cavity of all tibiae in the infection groups. All animals of the control group received the same procedure using a sham liquid of 10 μl sterile Tryptic Soy Broth (TSB). A closed reduction of the fracture was performed with 0.8 mm k-wire ostheosynthesis. The fascia and skin were sutured in a single-knot technique.

#### Growth factor application

All animals received second surgery five weeks after the first surgery. The skin incision was placed over the scar of the previous suturing. The k-wire was carefully removed and the medullar cavity was irrigated with sterile saline. All animals of the control and infected control group received an intramedullary injection of a control fluid of 30 μl sterile saline at the fracture site. The other infected animals received an intramedullary injection of 30 μg rhBMP-7 (1 μg/μl in sterile saline, Olympus, USA) or 25 μg rhBMP-2 (1 μg/μl in sterile saline, Medtronic, USA) respectively. A new k-wire was applied for osteosynthesis and the skin and fascia were sutured in a single-knot technique.

#### Body weight and body temperature

Rectal body temperature was measured and body weight was determined with a precision scale on post-operative days 0, 7, 14, 35, 42 and 70. Further indications for local or systemic infections were evaluated.

#### Sacrifice

The animals were sacrificed with CO_2_ in a sedation box. The right tibiae of the hind legs were dissected under sterile conditions. The entire soft tissue was removed from bones.

#### Biomechanical testing

**Fig. 1:**
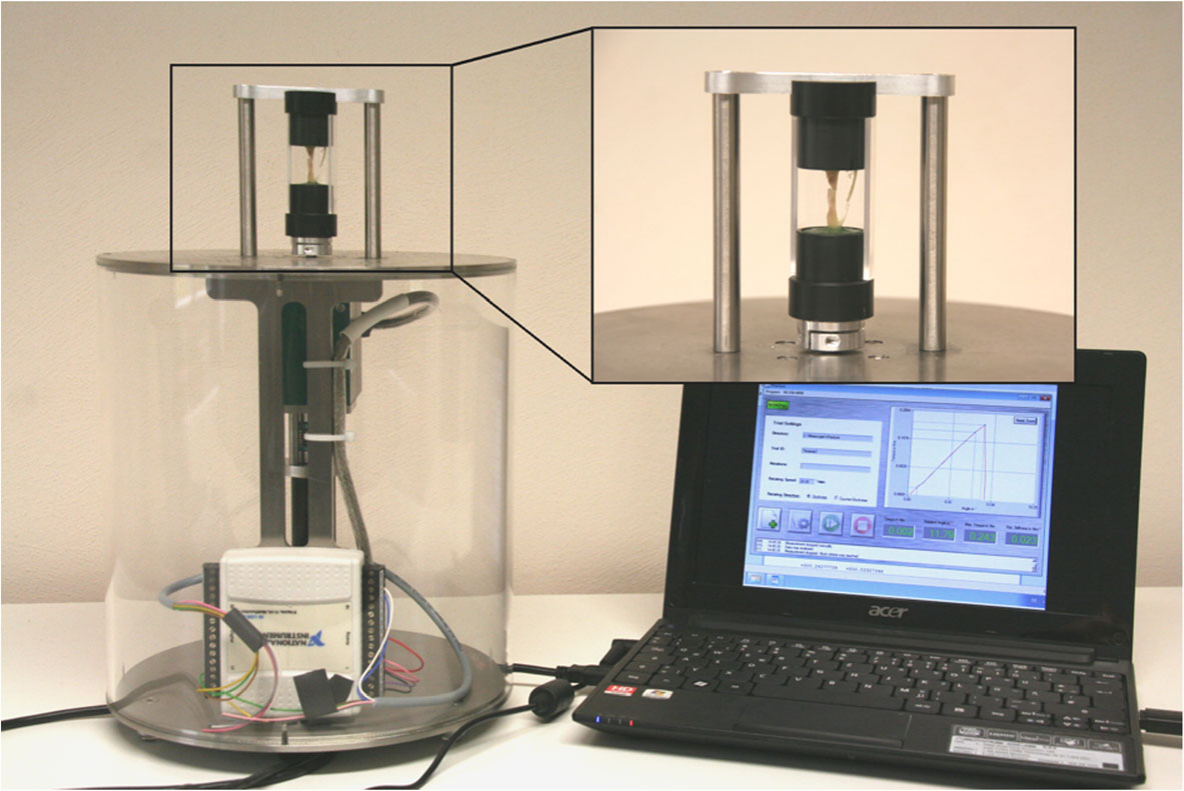
Biomechanical torsional testing machine with bone segment fixed in vertical direction by two embedding molds (detail view). A linear, constant rotation speed of 10°/min was applied to measure the resultant torque needed to induce re-fracture

At the 10 weeks endpoint the intramedullary implants were carefully removed (Control group: 9 samples; infected control group: 9; rhBMP-7 group: 8; rhBMP-2 group: 7). Both tibiae were dissected free from soft tissue for biomechanical torsional testing. After dissection of the bones, the proximal and distal ends were placed into two embedding molds (Technovit 4071, Heraeus Kulzer GmbH, Germany) while using a fixture device to avoid any preloading of the bones prior to testing. The lower embedding mold was connected to a pivoted axis while rotation of the upper mold was restrained (Fig. 1). A linear, constant rotation (10°/min) was applied by the biomechanical testing device while the resultant torque was recorded (8661–4500-V0200, Burster, Germany) until bony fracture occurred. For biomechanical testing a comparison with the contralateral tibiae of each animal was done.

#### Micro-CT

The fractured tibiae of all animals were scanned with the SkyScan 1076 in-vivo micro-computertomograph (Brucker micro-CT, Belgium) at the endpoint (10 weeks after fracture). The tibiae were scanned with an isotropic pixel size of 18 μm and energy settings of 100 kV (voltage), 400 ms (exposure time) and 100 μA (current) through a 1.0-mm aluminium filter. Image reconstruction, including ring artifact reduction (20/20), beam hardening correction (30%) and smoothing (1/10) was performed using the SkyScan NRecon software (v. 1.6.9.8, Brucker microCT, Belgium).

Qualitative evaluation of the datasets was performed by simultaneously viewing multiple orthogonal slices in SkyScan DataViewer (v. 1.4, Brucker microCT, Belgium). Additionally, new bone formation at the fracture site, bridging of the fracture and bone remodeling were analyzed semi-quantitatively adopted from the scoring system of Lane and Sandhu and given points from 0 (absent), 1 (25%), 2 (50%), 3 (75%) to 4 (100%) [21]. Hence, the highest possible score was 12 points. The scoring system was used as “bone-healing-score”. The modified score of An and Friedman was used to determine the grade of osteitis at the endpoint [22, 23]. The score of An and Friedman is a radiographic scoring system for assessing the development and progression of osteomyelitis in a rabbit model. This score was used in the study as “bone-infection-score”. Seven characteristic parameters were analyzed: periosteal reaction (1), osteolysis (2), soft tissue swelling (3), deformity (4), general impression (5), sequester formation (6) and spontaneous fracture (7). For evaluation of the parameter 1 to 5, the tibia was divided into three regions of interest (ROI) – proximal epiphysis, diaphysis and distal epiphysis, and a score from 0 (absent), 1 (mild), 2 (moderate) to 3 (severe) was given to each ROI. For parameters 6 and 7 the whole tibia was graded with a score of either 0 (absent) or 1 (present). Therefore, the highest possible score was 47.

Bone morphometry of the 3D-dataset was performed using the software CTAn (v. 1.13.2.1., Brucker microCT, Belgium). The volume of interest (VOI) for each tibia was defined as the bone area in between 3.5 mm proximal and distal to the fracture line (400 slices) [24, 25]. The following parameters were used for bone morphometry of the VOI for each animal at the endpoint after 10 weeks: tissue volume (TV), bone volume (BV) and bone volume fraction (BV/TV).

#### Histology

The tibiae from each group were randomly harvested for histological evaluation at the endpoint after 10 weeks as previously described (Control group: 9 samples; infected control group: 7; rhBMP-7 group: 6; rhBMP-2 group: 7) [26]. Surrounding muscles and intramedullary implant were carefully removed. After fixation for 2 days in 10% normal buffered formaldehyde, dehydration was done in ascending concentrations of ethanol and followed by undecalcified embedding in methylmethacrylate (Technovit 9100, Heraeus Kulzer GmbH, Germany). Longitudinal sections in a sagittal plane were cut at 6 μm with a Leica SM 2500 s microtome (Bensheim, Germany) with a 40° stainless-steel knife. Different stains were used, including von Kossa/Safranin-O, Masson Goldner and Gram stain for microscopic visualization of mineralized bone, cartilage and *S. aureus* in the tissue and bone, respectively. Evaluation was performed with a Leica DM-RB microscope (Bensheim, Germany). Qualitative changes were first evaluated in representative slides depicting the fracture gap area. Further histological parameters were measured with an image analysis system (Zeiss KS 400, Germany) at a magnification of × 1.6 to calculate structural indices as described previously [26]. The region of interest was defined at 3.5 mm proximally and distally to the center of the fracture gap. The total diameter of the callus was included in the ROI. For quantitative histomorphometric analysis, the area of the total callus (Total callus area; [mm^2^]), the area of the periosteal callus (periosteal callus area; [mm^2^]), and the mineralized periosteal bone area of the periosteal total callus area (periosteal bone area/periosteal total area; [%]) were measured and compared between the four groups.

#### Statistics

Primary outcome measure was fracture torque in biomechanical analysis. Secondary outcome measures were the results from the bone-healing-score and the bone-infection-score, and the results from quantitative micro CT and histomorphometry. The sample size planning showed an effect size f of 0.82 and an actual power of 0.878 with a sample size of 6 animals per group. A final sample size of 9 animals per group was chosen to compensate drop-outs. The post hoc power analysis provided an effect size f of 0.71 and an actual power of 0.918 for the biomechanical evaluations as the primary end point. For descriptive statistics, mean and standard deviation (SD) were calculated for continuous, median, and interquartile ranges for ordinal variables. Group comparisons were performed using one-way analysis of variance (ANOVA) for independent samples. All tests were two-sided and a *p*-value ≤0.05 was considered significant. Statistical analysis was performed with SPSS software (v. 22.0, IBM Corporation, USA).

## Results

### Body weight and body temperature

No significant differences of body weight and body temperature were detected between the four groups. Body weight moderately decreased in the four groups during the first two weeks after surgery, but the animals continuously gained weight afterwards reaching normal weight again at the endpoint. Body temperature remained stable in all groups during the 10 weeks follow-up.

### Drop-outs

Three animals died at the first and second operation, respectively, due to complications with general anesthesia. Two animals were sacrificed because of a postoperative infected hematoma. Further 5 specimens had to be excluded due to technical problems during the preparation of the tibiae.

### Biomechanical testing

**Fig. 2:**
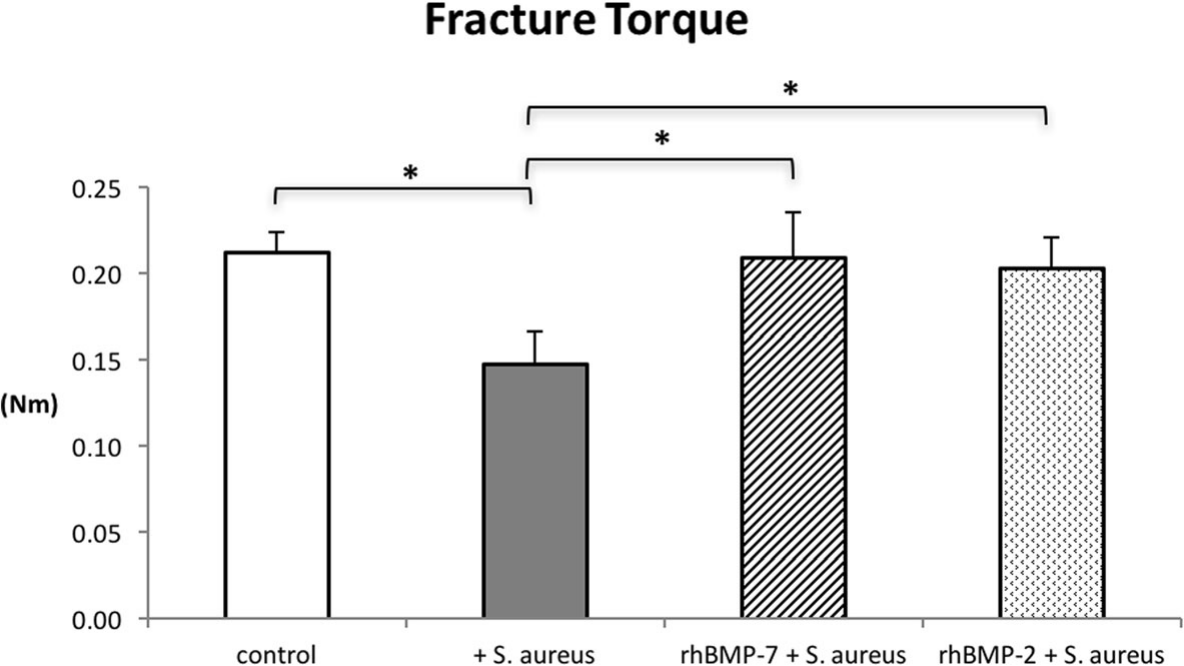
Mechanical testing results of the tibiae: the re-fracture torque (Nm) is significantly lower in the infected control group than in the other three groups at the endpoint. Lines with asterisk depict significant differences between groups (* *p* < 0.001)

The K-wires could be removed easily in all cases, with no differences detected between the groups. At the contralateral non-fractured side, all groups did not show statistically significant differences (*p* = 0.9). Average fracture torques (Nm) was 0.213 +/− 0.022 Nm in the control group, 0.227 +/− 0.087 Nm in the infected control group, 0.227 +/− 0.074 Nm in the rhBMP-7 group and 0.206 +/− 0.035 Nm in the rhBMP-2 group at the non-fractured side. At the fractured side, Fracture torques (Nm) in the rhBMP-2, rhBMP-7, and control group were significantly higher than in the infected control group (*p* < 0.001) (Fig. 2). The differences between the rhBMP-7 group and rhBMP-2 group were not statistically significant (*p* = 0.6) (Fig. 2). The fractured tibiae of the control group showed an average fracture torque of 0.212 +/− 0.012 Nm, the rhBMP-7 group an average fracture torque of 0.209 +/− 0.026 Nm and the rhBMP-2 group a fracture torque of 0.203 +/− 0.018 Nm compared to 0.147 +/− 0.019 Nm in the infected control group (Fig. 2).

### Micro-CT results

**Fig. 3:**
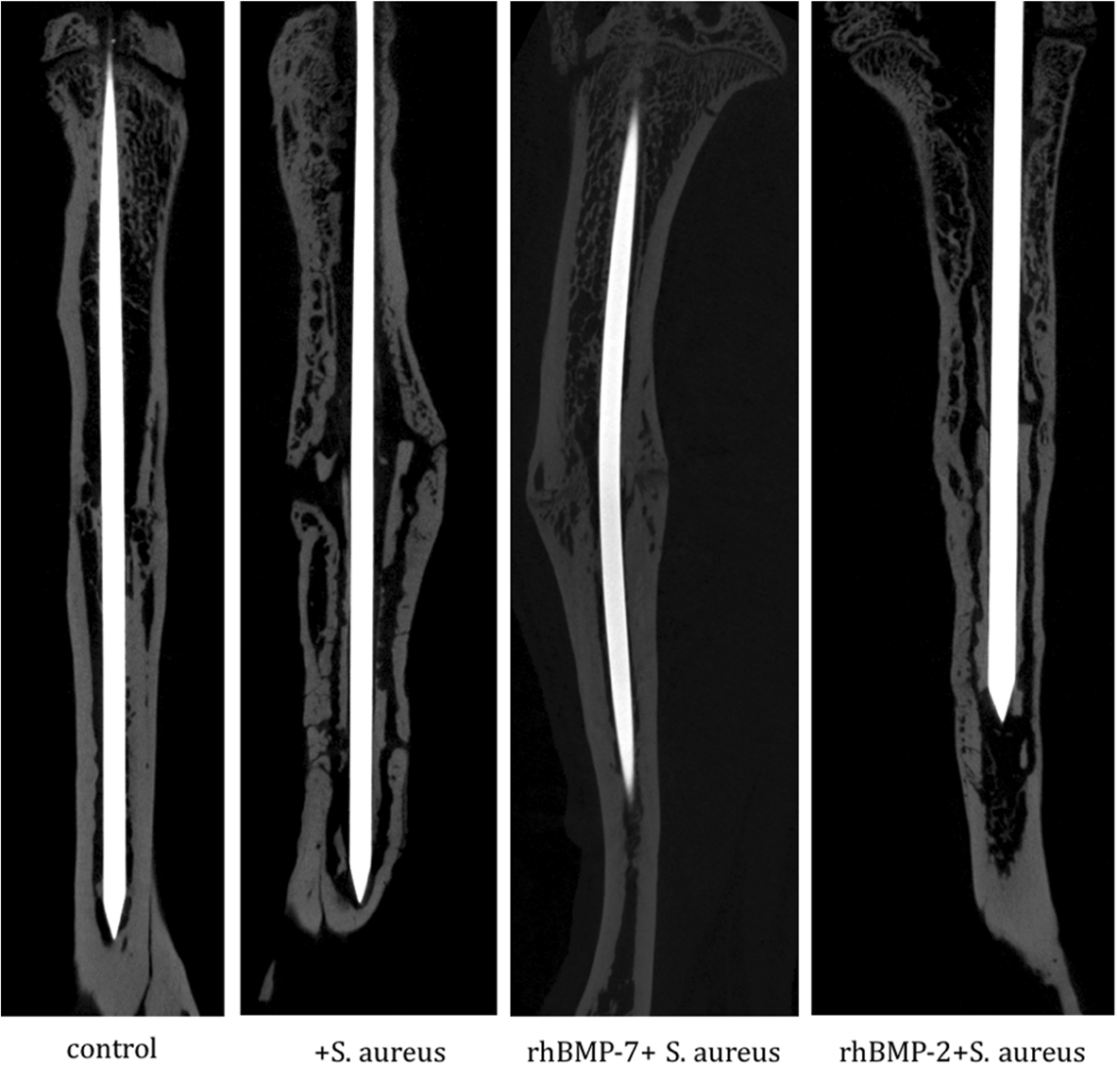
Micro computed tomography (micro-CT) of the right tibiae of Sprague-Dawley rats at the endpoint. Fracture, bacterial infection or sham infection, and intramedullary stabilization with titanium Kirschner wires was performed 10 weeks before; application of rhBMP-7, rhBMP-2 or sterile saline was done 5 weeks before analysis. Improved consolidation of the fracture gap is recognizable in the control group compared to the three infected groups. Considerable differences in callus formation between both rhBMP groups were not detected

Micro-CT pictures of the non-infected control group showed increased consolidation with complete bridging of the fracture gap compared with both rhBMP-groups and the infected control group at the endpoint (Fig. 3). Compared to the non-infected control group, all infected groups (infected control group (*S. aureus*), rhBMP-7 group (rhBMP-7 + *S. aureus*), rhBMP-2 group (rhBMP-2 + *S. aureus*)) showed increased hypertrophic callus formation. Most callus was visible in the infected control group. Callus formation showed no differences between the rhBMP-7 and the rhBMP-2 group (Fig. 3).

**Fig. 4:**
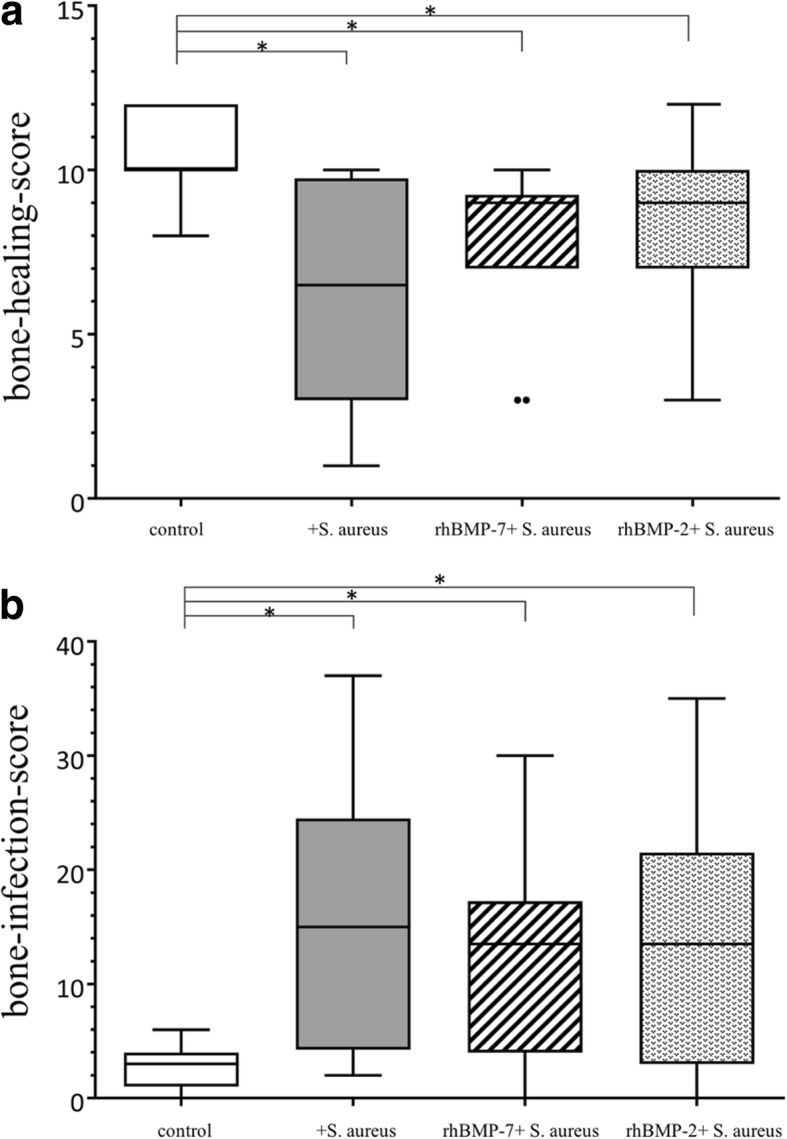
Outcome of (**a**) the bone-healing-score adapted from Lane & Sandhu with a maximal score of 12 points and (**b**) the bone-infection-score adapted from An & Friedman with seven characteristic parameters for a maximal score of 47 points. Both scores are significantly different between the control group and the three infected groups. Lines with asterisk depict significant differences between groups (* *p* < 0.05)

The non-infected control group presented a significantly higher bone-healing-score representative for best bone healing and bridging (*p* < 0.001) and a significantly lower bone-infection-score compared to all other three groups (p < 0.001). The scores of both rhBMP-groups (rhBMP-7 + *S. aureus*, rhBMP-2 + *S. aureus*) did not reach the level of the control group, but they showed better results than the infected control group (*S. aureus*) with fewer signs of osteitis, more bone formation and enhanced bone remodeling (Fig. 4a and b).

**Fig. 5:**
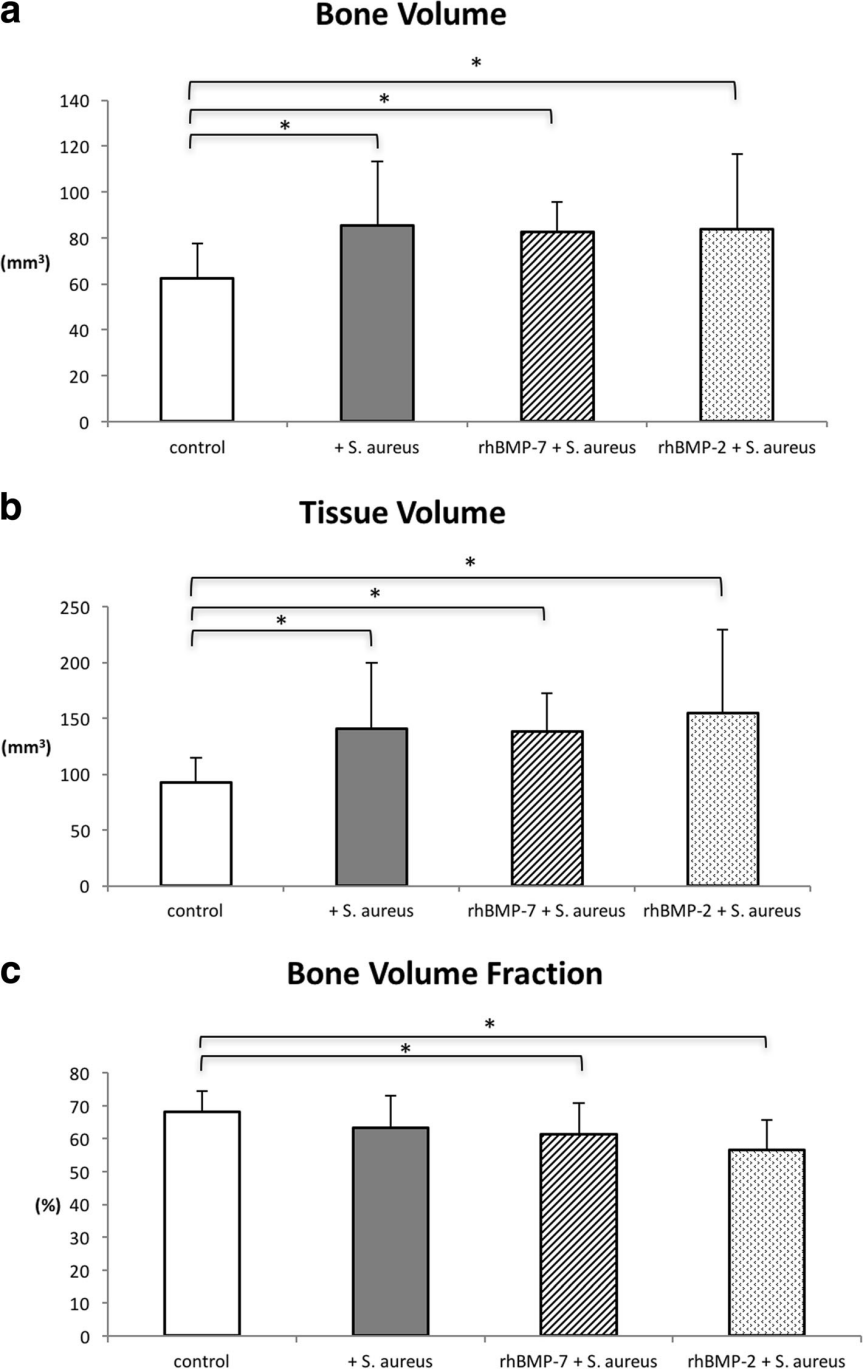
Quantitative micro-CT evaluation of bone volume (mm^3^) (**a**), tissue volume (mm^3^) (**b**) and bone volume fraction (%) (**c**) of the four groups at the endpoint. Lines with asterisk depict significant differences between groups (* *p* < 0.05)

Quantitative callus evaluation revealed that bone volume (BV) and tissue volume (TV) of the non-infected control group were significantly lower than of the infected groups (infected control group, rhBMP-7 group, rhBMP-2 group) (Fig. 5a and b). Additionally, the rhBMP-2 group and the rhBMP-7 group showed a significantly lower bone volume fraction compared to the control group. There were no statistically significant differences between the infected control group and both rhBMP-groups (Fig. 5c).

### Histology

**Fig. 6:**
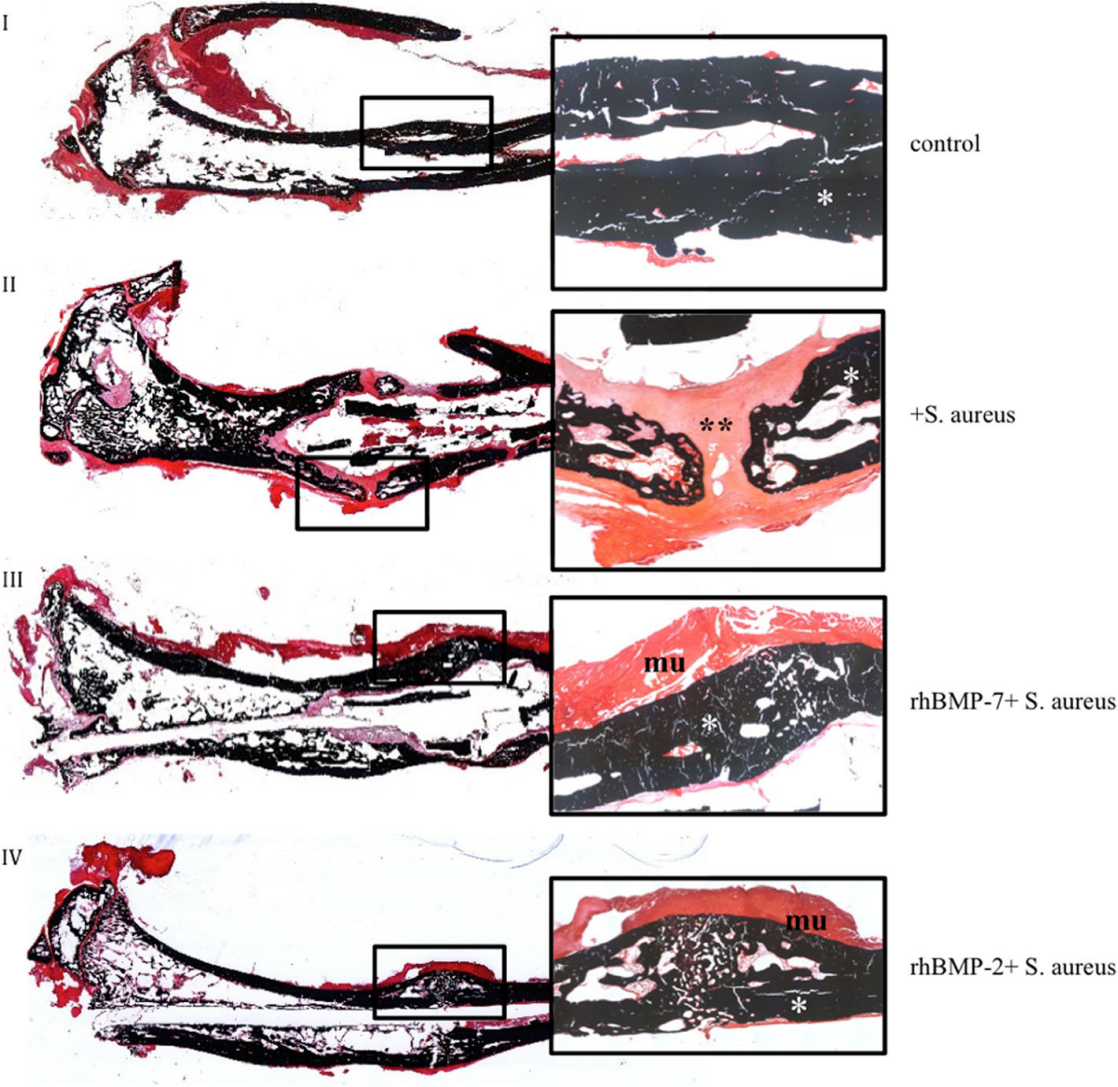
Overview and magnification (2.5×) of the fracture region stained with van Kossa/Safranin Orange (mineralized tissue: black; cartilage and fibrous tissue: red): In the non-infected control group the callus is mineralized (*). No fracture healing is visible in the infected control group (+*S. aureus*) with fibrous tissue and cartilage (**) filling the gap. Bone healing of the fracture is increased in both rhBMP groups with less fibrous tissue and less cartilage filling. I) control group, II) infected control group (+*S. aureus*), III) rhBMP-7 group (rhBMP-7 + *S. aureus*) and IV) rhBMP-2 group (rhBMP-2 + *S. aureus*); mineralized tissue: *; fibrous tissue and cartilage: **; muscles: mu

**Fig. 7:**
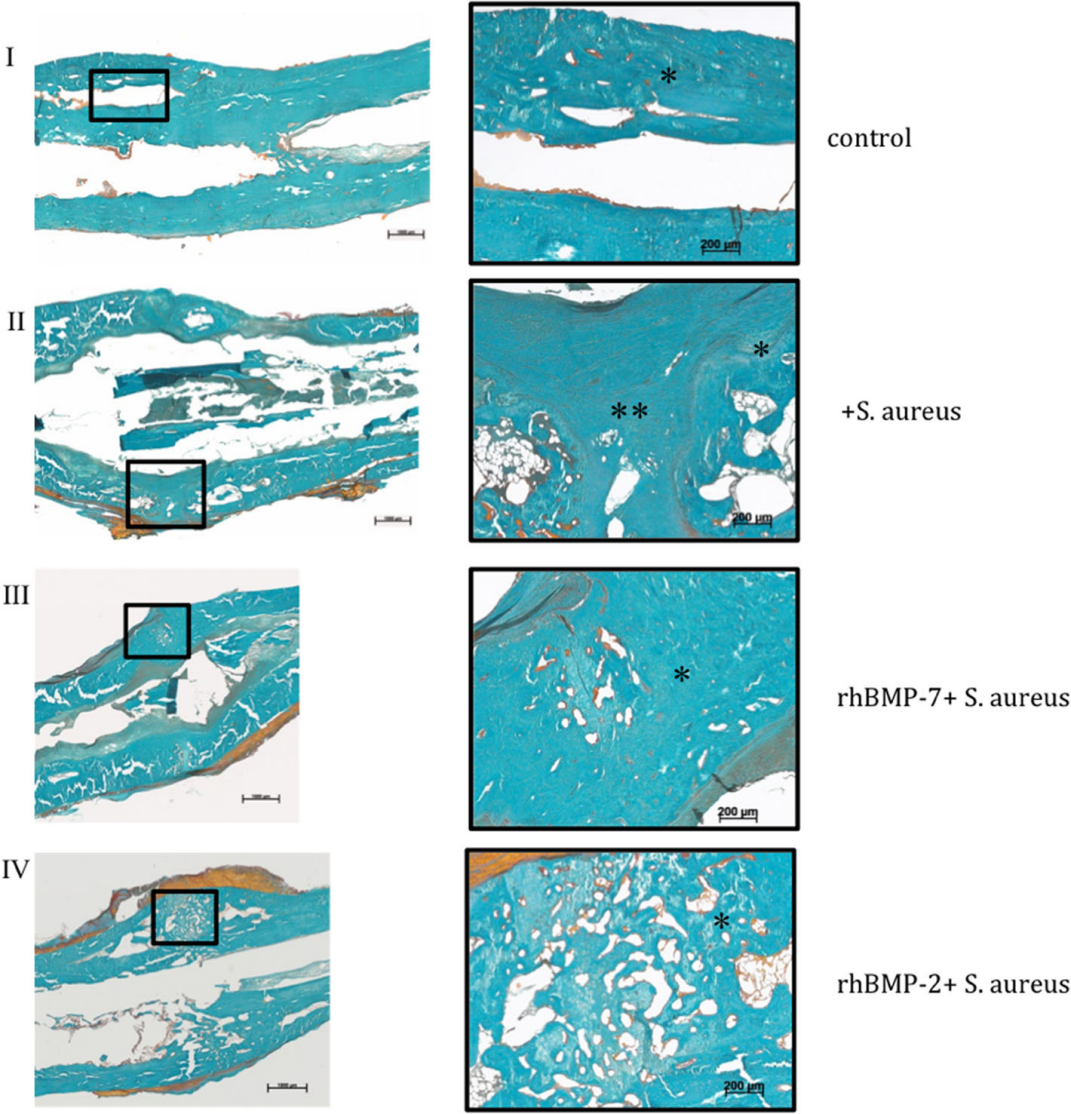
Magnifications (2.5× and 10×) of the fracture region stained with Masson Goldner (mineralized tissue: turquoise; cartilage and fibrous tissue: green; nuclei: dark brown; muscles: brick-red): In the non-infected control group the callus is mineralized (*). No fracture healing is visible in the infected control group (+*S. aureus*) with fibrous tissue and cartilage (**) filling the gap. Bone healing of the fracture is increased in both rhBMP groups with less fibrous tissue and less cartilage filling. I) control group, II) infected control group (+*S. aureus*), III) rhBMP-7 group (rhBMP-7 + *S. aureus*) and IV) rhBMP-2 group (rhBMP-2 + *S. aureus*); mineralized tissue: *; fibrous tissue and cartilage: **

**Fig. 8:**
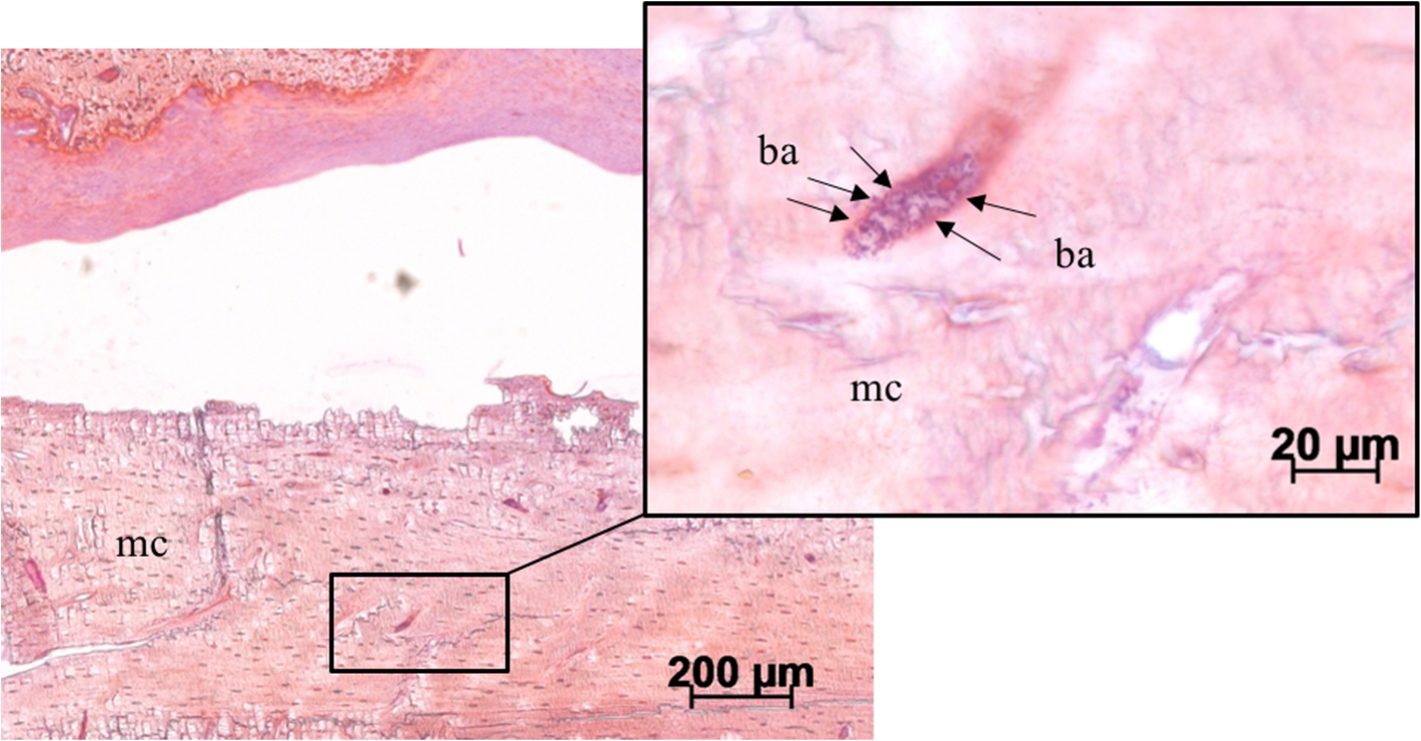
Bacterial infection visible in 5× and 40× magnification of the cortex region stained with Gram (bacteria: dark purple; mineralized tissue: pink): *S. aureus* were found accumulated in the original cortex. Mineralized cortex: mc; bacteria: ba

Qualitative evaluation of histological slides showed good callus formation and progressed bone remodeling with few connective tissue in the non-infected control group. In the infected control group, more fibroblasts and cartilage were observed in the fracture region (Figs. 6 and 7: II). Both rhBMP groups showed partially remodeled fractures at the endpoint with only moderately remaining cartilage or fibrous tissue (Figs. 6 and 7: III and IV). Gram stain showed *S. aureus* in the orginal cortex and cancellous bone in all specimens of the three infected groups (Fig. 8). The bacteria were encapsulated in the cortex fragments. *S. aureus* was not found in the newly formed woven bone in all three infected groups. There were no significant differences between the three infected groups. Additionally, the three infected groups showed a second cortex in the histology. Significant differences between these groups were not detectable.

**Fig. 9:**
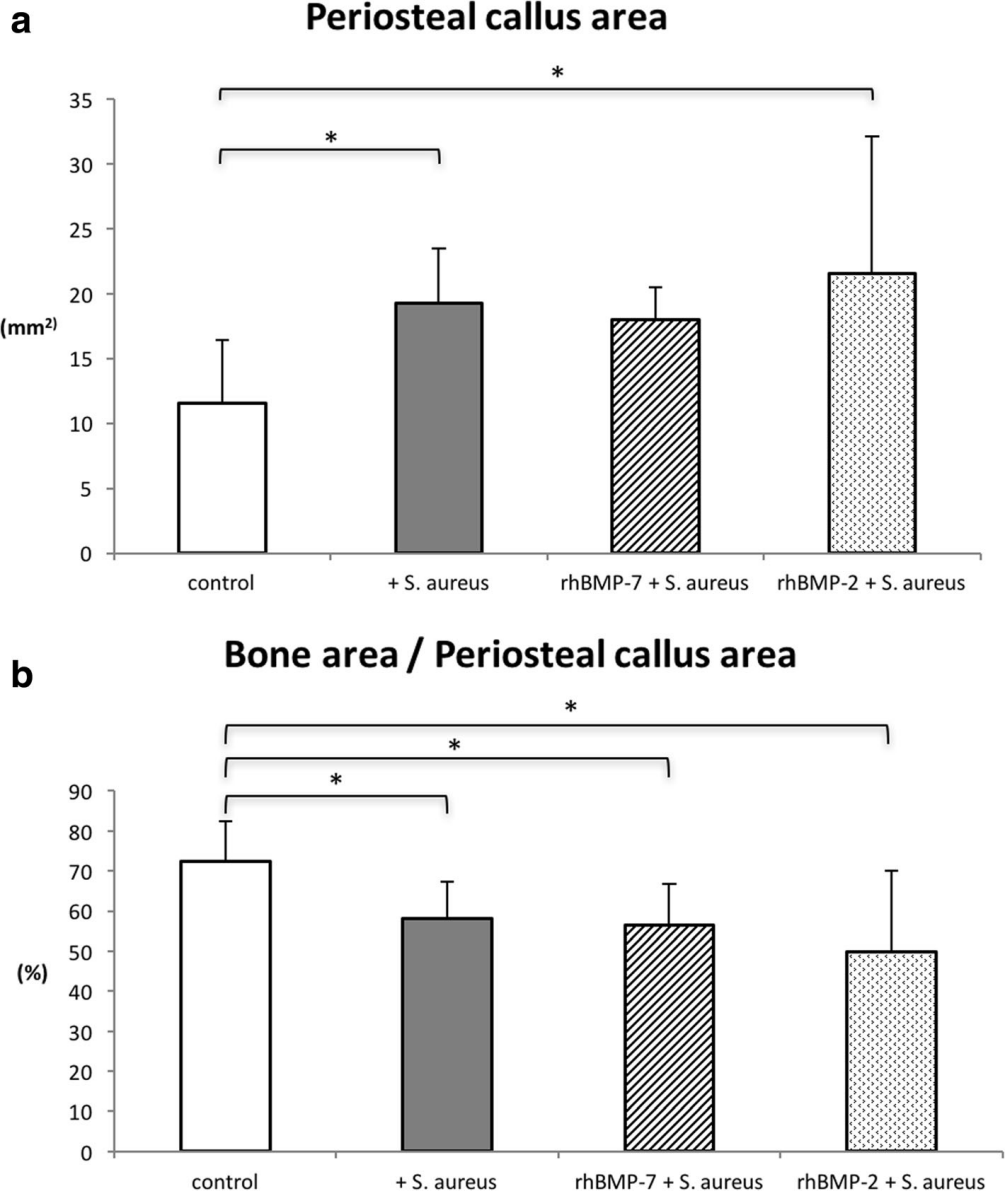
Histomorphometric evaluation of (**a**) periosteal callus area within the fracture gap (mm^2^) and (**b**) mineralized bone tissue in the periosteal callus (%) between the groups at the endpoint. Lines with asterisk depict significant differences between groups (* *p*≤0.05)

Histomorphometric analyses of the periosteal callus area showed significant differences in the infected control group (*p* = 0.009) and the rhBMP-2 group (*p* = 0.037) compared to non-infected controls (Fig. 9a). Analysis of callus composition still revealed significantly less mineralized bone tissue in the periosteal callus in the rhBMP-2 (*p* = 0.005) and rhBMP-7 group (*p* = 0.05) compared to non-infected controls (Fig. 9b). Infected controls and both rhBMP groups did not show significant differences (*p* = 0.8 (rhBMP-7) and *p* = 0.3 (rhBMP-2)).

## Discussion

In this study, we have evaluated the effect of recombinant human bone morphogenetic proteins − 7 and − 2 for the treatment of delayed osseous union secondary to bacterial osteitis in our animal model using micro-CT examinations, qualitative histology, histomorphometric evaluations, and biomechanical investigations. To our knowledge this is the first study, which compared recombinant human bone morphogenetic proteins − 7 and − 2 in an in vivo animal model of delayed osseous union in the situation of an ongoing bacterial bone infection.

We have chosen rhBMP-7 and -2 in our study setting, because these growth factors have been established clinically in the treatment of non-union [15, 27–29]. Giannoudis et al. [29] could show a synergistic effect of autograft and bone morphogenetic protein − 7 in the therapy of atrophic humeral, femoral and tibial non-union with a healing rate of 100% in 45 patients. Govender et al. [15] investigated rhBMP-2 in the treatment of open tibial fractures and showed better fracture- and wound-healing and reduced infection rate. Similar effects have been described by others [30, 31]. Significant differences in the effectiveness of both factors or synergistic effects have not been described.

According to micro-CT-scans and histology, treatment with rhBMPs was unable to achieve a complete union in the situation of infection during the follow-up period, but we could find positive effects on bone healing in biomechanical evaluations as well as in the semi-quantitative bone-healing-score and in callus histology. Biomechanical investigations and scoring of the micro-CT images revealed significantly more stability and healing in the infected rhBMP groups equal to non-infected controls if compared to the infected control group. Thus, the findings indicate that rhBMP treatment is possibly able to improve bone healing in the setting of infection, although it has no direct effect on the infection with *S. aureus* [32]. In this regard, our investigations supported the results in the literature [33, 34]. All infected groups, no matter if rhBMP therapy was added or not showed significantly increased mineralized callus (BV) and soft-tissue callus (TV), but a significantly decreased bone volume fraction (BV/TV) in quantitative micro-CT evaluation as well as larger periosteal callus areas and smaller mineralized periosteal bone areas of the periosteal total callus area in histology. This may be interpreted as a hypertrophic callus, explaining less stability compared with the non-infected control group. In agreement with other studies, Schmidmaier et al. [28] and Bode et al. [35] postulated that an infection or an instable fixation are the cause of a hypertrophic non-union formation. They recommend repeated debridements in case of infected, especially open fractures, with stable osteosynthesis depending on the type of the fracture to prevent non-union. In the present study, histology further showed some qualitative differences of the callus. In rhBMP specimens, we saw increased signs of fracture remodeling with only moderately remaining cartilage and fibrous tissue. These changes seemed less pronounced in infected controls without rhBMP treatment. In contrast to the study of Chen et al. [33, 34], which used a rat femur model with a segmental chronically infected bone defect, we were not able to achieve significantly more newly mineralized callus compared to infected controls in our animal model. A possible reason for this better mineralization might be the additional antibiotic treatment in the study of Chen et al.

As a limitation of our study, we did not analyze longer follow-up times, so further effects might have been detectable after the analyzed duration of 5 weeks after rhBMP treatment. Furthermore, we were not able to specifically identify or quantify the underlying mechanisms for the increased stability, possibly due to technical limitations as the bone specimen had to be collected, removed from soft tissue, further prepared and fixed before cutting into 6 μm sections for histological slides. Hence, semi-quantitative histological assessment of the callus architecture was not possible. Nevertheless, signs of partial fracture remodeling were mainly found in rhBMP specimen contrary to more fibroblasts and remaining cartilage in infected controls.

Clinical studies such as the BESTT study (BMP-2 Evaluation in Surgery and Tibial Trauma) have presented both an accelerated healing process and a lower infection rate after treatment with rhBMP-2 [15]. Similarly successful results were obtained by a combined two-step non-union therapy with rhBMP-7 after osteomyelitis [36]. However, it should be taken into consideration that clinical routine therapy of bone infections also involves debridement of necrotic bone, soft tissue management and antibiotic therapy [4, 37]. These procedures lead to reduction of bacterial load and open the way for osteoprogenitor cells from the periosteum, blood vessels and soft tissue into the defect [38, 39]. Similar procedure was used in the rhBMP-2 and rhBMP-7 studies in a rat model of Chen et al. [33]. In the experimental setting of our study only intramedullary irrigation with sterile saline, rhBMPs application and k-wire replacement were performed, which will have maintained a high bacterial burden in the bone. Most clinical cases of unsuccessful non-union treatment reveal subclinical infection [36]. This clinical experience, together with the findings of the current animal study suggest that an ongoing bone infection will hamper bone healing with potentially reduced effectiveness of the rhBMPs. Further in-vivo studies might help to get further information on this topic. RhBMP application in an animal model together with differently concentrated bacterial suspensions and without could be analyzed, to determine potential bacterial-dose-dependent effects. Also adverse effects have been described in the literature for rhBMP therapy [40–43] but were not found in the present animal study at least in the short term, as we did not find rhBMP associated side-effects or complications such as heterotopic ossifications or induction of neoplasms [42, 43].

Future studies should investigate later time-points of bone healing in order to compare the biomechanical, histological and radiological results with the present study. Other growth factor application systems as well as other antibacterial or bone stimulating substances and osteosynthesis techniques [44] could also be analyzed with the present animal model considering their effectiveness in case of infection. The long-term goal would be to establish a material or a combination of materials and techniques that have osteoinductive and osteoconductive but also antimicrobial effects to provide bone healing within a short operation and healing period.

## Conclusions

We could establish a rat model of delayed osseous union secondary to experimental fracture and bone infection with biomechanical, histological, and micro-CT evaluation after treatment with rhBMP-2 and rhBMP-7 compared to infected and non-infected controls. The induced bone infection caused quantitatively more callus tissue in all infected groups. In the infected control group callus was biomechanically less stable and showed less bridging. RhBMP treatment increased biomechanical stability of the callus without significant differences between rhBMP-2 and rhBMP-7. Our results demonstrate that rhBMP treatment can improve bone-healing in the situation of a chronic bone infection supporting clinical use in complicated infected non-unions.

## Abbreviations

BV: bone volume
CFU: Colony-Forming Unit
CT: Computer Tomography
rhBMP: Recombinant human Bone Morphogenetic Protein
ROI: Region of interest
*S. aureus*: *Staphylococcus aureus*
TSB: Tryptic Soy Broth
TV: Tissue volume
VOI: Volume of interest
